# Sarcopenia and Walking Recovery in Older Adults: A Mini-Review of Evidence and Rehabilitation Implications

**DOI:** 10.3390/jcm15155904

**Published:** 2026-07-29

**Authors:** Gulnara D. Otepova, Mohammad Khoshraftar, Saule T. Tazhbenova, Perizat Z. Aitmaganbet, Lyudmila S. Yermukhanova, Maral G. Nogayeva, Alireza Afshar

**Affiliations:** 1 Institute of Preventive Medicine Named After Academician E.D. Dalenov, Non-Profit Joint-Stock Company Astana Medical University, Astana 010000, Kazakhstan; g.otepova@gmail.com; 2 Student Research Committee, Bushehr University of Medical Sciences, Bushehr 7514633341, Iran; khoshraftarm.mk@gmail.com; 3 Department of Public Health and Healthcare with the Course of Nursing, Marat Ospanov West Kazakhstan Medical University, Aktobe 030000, Kazakhstan; saule.tazhbenova@zkmu.kz (S.T.T.); perizat.a@zkmu.kz (P.Z.A.); yermukhanova@zkmu.kz (L.S.Y.); 4 Department of Rheumatology, Asfendiyarov Kazakh National Medical University, Almaty 050012, Kazakhstan

**Keywords:** sarcopenia, rehabilitation, gait, muscle strength, exercise therapy

## Abstract

Walking recovery is a central goal of geriatric rehabilitation, but it is shaped by multiple interacting factors rather than by muscle impairment alone. This mini-review examines whether sarcopenia-related weakness, low muscle quality, and reduced physical reserve identify a clinically important vulnerability phenotype relevant to delayed gait recovery in older adults after hospitalization, hip fracture, surgery, or cardiac procedures. We summarize current consensus definitions of sarcopenia, explain why muscle strength and physical performance are often more clinically informative than muscle mass alone, and review the mechanistic pathways that may connect sarcopenia with impaired walking, including anabolic resistance, inflammation, disuse, neuromuscular remodeling, trunk weakness, and cognitive–emotional modifiers. Across community, orthogeriatric, post-acute, and cardiac rehabilitation settings, sarcopenia markers are generally associated with slower gait or poorer attained mobility; however, findings for change in mobility are inconsistent, and most available studies are observational, heterogeneous, or indirect. Sarcopenia should therefore be interpreted as one potential marker of reduced reserve and mobility risk rather than as a determinant of walking recovery or evidence that sarcopenia-aware rehabilitation improves outcomes. Future work should use more consistent diagnostic frameworks and test whether a sarcopenia-aware rehabilitation integrating early strength assessment, individualized nutrition care, and multicomponent gait rehabilitation improve walking outcomes in medically complex older adults beyond current standards of care.

## 1. Introduction

Recovery of walking is a core outcome in geriatric rehabilitation because even small changes in gait speed are linked to survival, disability risk, discharge destination, and broader functional recovery in older adults [1,2]. Walking recovery is also inherently multifactorial, because safe ambulation depends on muscle strength, balance control, cardiorespiratory capacity, pain burden, cognition, motivation, and the clinical environment in which rehabilitation occurs [3,4,5]. For that reason, sarcopenia should be considered one clinically important and potentially modifiable contributor to delayed gait recovery, rather than the sole explanation for mobility loss after hospitalization, fracture, or surgery [5,6,7].

Older adults who do not recover walking may have sarcopenia-related weakness or poor physical performance in addition to orthopedic, cardiopulmonary, neurological, cognitive, and other determinants of mobility. Sarcopenia is a muscle disease characterized by low strength, low muscle quantity or quality, and impaired physical performance, but its contribution to an individual patient’s walking limitation cannot be separated reliably from these coexisting factors using the available observational evidence [5,6,7,8,9,10]. The current consensus literature is important here. EWGSOP2 places low muscle strength at the forefront of diagnosis, recommends muscle quantity or quality to confirm sarcopenia, and uses poor physical performance to grade severity. The revised AWGS framework similarly incorporates low strength and/or repeated chair stand performance together with muscle mass and performance measures, while the Sarcopenia Definitions and Outcomes Consortium has argued from outcomes-based analyses that weakness and slowness deserve central diagnostic weight because lean mass alone is not consistently associated with clinically relevant outcomes [7,11]. These definitional shifts matter for rehabilitation because walking recovery depends less on the static amount of muscle tissue than on the ability of muscle to generate force, power, and coordinated movement under real-world task demands. Accordingly, failure to regain community ambulation may reflect disease burden together with reduced gait reserve associated with sarcopenia, although the relative contribution of each factor cannot be determined from current observational evidence [7,8,9,10,12].

This issue is not marginal in rehabilitation populations. Sarcopenia is highly prevalent in geriatric rehabilitation inpatients, with reports exceeding half of admissions depending on the diagnostic method, and it is common in groups in whom walking recovery is a dominant clinical target, including older adults after hip fracture and older patients entering inpatient cardiac rehabilitation after cardiac procedures [7,13,14]. Systematic review data in hip fracture populations suggest a sarcopenia prevalence around 44%, and most included studies report poorer postoperative functional recovery in sarcopenic than in non-sarcopenic patients. In older cardiac patients undergoing inpatient cardiac rehabilitation, recent prospective data found sarcopenia in roughly 35% at baseline, with strong correlations between sarcopenia screening scores and poor functional capacity measures such as handgrip strength, SPPB, and 6 min walk distance. In older in-hospital rehabilitation cohorts, even a probability-based sarcopenia construct has been associated with worse Barthel Index performance and lower walking ability at discharge [14].

In this review, gait reserve is a practical heuristic, not a validated diagnosis or billing category. It denotes the task-specific margin between walking capacity under a standardized low-complexity condition and performance when speed, endurance, postural control, or attention demands increase. A simple reference condition can be usual-pace straight-path walking over 4 m or another locally standardized short course; the 6 min walk test, Short Physical Performance Battery (SPPB), or Timed Up and Go (TUG) can supplement the reference profile when feasible [1,7,15,16]. EWGSOP2 uses gait speed ≤ 0.8 m/s, SPPB ≤ 8, or TUG ≥ 20 s to indicate poor physical performance, whereas AWGS 2019 uses gait speed < 1.0 m/s, SPPB ≤ 9, or a five-chair-stand time ≥ 12 s. These cutoffs classify physical performance within their respective sarcopenia frameworks; they are not validated thresholds for gait reserve [7,15].

Clinical measurement should therefore focus on within-person change between a standardized reference task and one or more prespecified challenges, such as fast-pace walking, dual-task walking, repeated turns, obstacle negotiation, or prolonged ambulation. For example, dual-task gait-speed cost can be reported as 100 × (single-task speed − challenged speed)/single-task speed, while also reporting the absolute speeds, assistive device, task instructions, and safety support [16]. No universal percentage separates preserved from reduced gait reserve, and a single challenged trial should not determine treatment eligibility. Serial change under the same protocol is more defensible than applying an unvalidated cutoff, particularly during acute recovery.

Gait reserve also differs from adjacent constructs. Mobility capacity describes what a person can do under standardized conditions; functional reserve is a broader excess capacity across organ systems and activities; and physical resilience concerns resistance to, or recovery from, functional decline after a health stressor [17]. Gait reserve is narrower: it is the adaptable margin within walking performance when task demands change. Sarcopenia may reduce this margin through weakness, impaired power, disuse, inflammation, neuromuscular remodeling, trunk instability, fear, or cognitive load, but these mechanisms remain contributory rather than exclusive [5,18,19,20].

The purpose of this mini-review is to examine whether sarcopenia-related weakness, low muscle quantity or quality, and reduced physical performance define a clinically relevant vulnerability phenotype within the broader determinants of walking recovery [5,6,7]. We use the single term sarcopenia-aware rehabilitation for a hypothesis-generating process in which clinicians identify probable or confirmed sarcopenia, interpret walking limitation alongside strength and muscle measures, and consider whether usual individualized rehabilitation requires additional attention to resistance or power training, balance, and task-specific gait practice, while independently identified malnutrition or nutrition risk is managed through guideline-concordant individualized care [21,22,23]. The term does not denote a validated protocol, a replacement for comprehensive geriatric or frailty assessment, or evidence that sarcopenia-specific treatment improves gait. Observational findings are interpreted as prognostic or risk-stratification evidence unless a study directly tested treatment efficacy [8,24,25].

## 2. Literature Identification and Interpretive Approach

Because this article is a narrative mini-review, the literature was assembled purposively rather than through a prospectively registered systematic review protocol; nevertheless, we sought to describe the search, selection, and appraisal approach transparently in accordance with established quality principles for narrative reviews [26]. PubMed/MEDLINE was searched from database inception through 10 June 2026, supplemented by reference list screening of relevant consensus statements, systematic reviews, and meta-analyses. Retrieved references were managed and organized using EndNote, version (V21, Clarivate, London, UK). Search combinations included sarcopenia, probable sarcopenia, muscle strength, muscle quality, gait, walking, mobility, rehabilitation, hip fracture, post-acute care, cardiac rehabilitation, resistance exercise, and nutrition. We prioritized international consensus definitions, systematic reviews and meta-analyses, randomized trials, and prospective or rehabilitation-based cohorts involving adults aged 60 years or older and reporting walking, physical performance, functional, or rehabilitation outcomes. Case reports, animal studies, conference abstracts without sufficient outcome data, and studies without a mobility-relevant outcome were not used to support clinical inferences.

Selection was guided by relevance to four settings: community-dwelling older adults, hip fracture and orthogeriatrics, geriatric post-acute rehabilitation, and cardiac rehabilitation. Study identification and selection were not performed independently by two reviewers, a PRISMA flow diagram was not generated, and no new meta-analysis was undertaken. Consequently, the search should be regarded as transparent but not exhaustive, and omission and selection bias remain possible [26]. Evidence from community-dwelling populations was used to inform biological plausibility and intervention design when direct post-acute evidence was unavailable, but it was not treated as direct proof of effectiveness in medically complex rehabilitation inpatients [10,24,27,28].

Evidence was appraised at the setting-and-claim level across four explicitly defined domains adapted from established evidence certainty principles: design-related risk of bias, including randomization, prospective ascertainment, attrition, confounding control, and validity of measurements; consistency across studies; directness to medically complex rehabilitation populations; and precision of reported estimates [29]. Moderate confidence indicates multiple reasonably consistent studies without a dominant critical limitation. Low-to-moderate confidence indicates a replicated signal constrained by observational design, indirectness, heterogeneity, or imprecision. Low confidence indicates sparse, inconsistent, substantially indirect, or heavily confounded evidence [29]. These categories represent structured narrative judgments rather than formal GRADE ratings because the review was not prospectively registered, study selection was not duplicated, and formal study-level risk-of-bias assessment using design-specific instruments was not undertaken [26,29,30]. Prognostic or risk stratification confidence was evaluated separately from confidence in treatment efficacy because an exposure may predict an outcome without demonstrating that treatment directed specifically at that exposure changes the outcome [9,10,29].

## 3. Sarcopenia as a Mobility-Limiting Rehabilitation Phenotype

Sarcopenia is no longer adequately understood as loss of muscle mass alone. EWGSOP2 defines low muscle strength as the primary parameter, uses low muscle quantity or quality to confirm the diagnosis, and classifies poor physical performance as severe sarcopenia. AWGS 2019 retains a similarly multidimensional approach, while SDOC has emphasized that a definition anchored in weakness and slowness may have better clinical validity for disability-related outcomes than one centered on lean mass [7,15]. This evolution is directly relevant to gait recovery because walking is a performance task, not a body composition task. A patient may have relatively preserved lean mass but still exhibit poor force production, low power, impaired balance reactions, or slowed walking that place recovery at risk [6,7].

At the same time, diagnostic heterogeneity remains a major barrier. Recent comparative work shows poor agreement among contemporary sarcopenia definitions, with low concordance between EWGSOP2 and SDOC in community cohorts, and scoping review evidence indicates that current definitions have not been validated evenly across outcome domains or populations [31,32]. The problem becomes even more tangible in rehabilitation wards. In the RESORT cohort, probable sarcopenia prevalence varied dramatically depending on whether handgrip strength or chair stand testing was used, and handgrip strength predicted institutionalization and mortality whereas chair stand testing did not. These findings suggest that the same patient may be labeled differently depending on the diagnostic pathway, and that some tools are more clinically informative than others in medically complex rehabilitation patients [33].

From a rehabilitation standpoint, it is also essential to distinguish primary, secondary, and acute sarcopenia. Primary sarcopenia refers to age-related muscle failure in the absence of another dominant cause. Secondary sarcopenia arises when chronic disease, inflammation, reduced activity, or malnutrition accelerate decline. Acute sarcopenia refers to rapid losses in muscle function and mass after hospitalization, surgery, trauma, or severe illness, and this category is particularly relevant to walking recovery because it develops precisely in the period when rehabilitation is trying to restore mobility [33,34]. Reviews of incident sarcopenia in hospitalized older adults have reported newly developed sarcopenia in roughly 12% to 38.7% of patients after hospitalization, and a recent meta-analysis estimated pooled acute sarcopenia incidence during hospitalization at about 18%, with higher rates in trauma and critical care settings [33,35].

Why does this matter for gait rehabilitation more than mass-based thinking would suggest? Because multiple lines of evidence indicate that strength, slowness, and muscle quality are the components most tightly tied to outcomes that matter clinically [6,25]. SDOC-derived work found that weakness and slowness predicted incident mobility limitation, while recent comparative analyses concluded that lean mass was not consistently associated with adverse outcomes. In parallel, the muscle quality literature has documented substantial heterogeneity in definitions and measurement approaches, but also growing agreement that parameters such as intramuscular fat infiltration, echo intensity, and phase angle may reflect the functional health of muscle more meaningfully than mass alone. For walking recovery, this makes sarcopenia best understood as a rehabilitation phenotype marked by low reserve, low adaptability, and high vulnerability to transactional stressors such as pain, fear, fatigue, and environmental challenge [7,25,36].

In this review, EWGSOP2 and AWGS 2019 are treated as parallel operational frameworks rather than as hierarchically ranked definitions. We retain EWGSOP2 when interpreting studies that applied its diagnostic sequence and thresholds, and we retain AWGS 2019 when source studies used AWGS criteria or when discussing populations for which its Asian-specific thresholds were developed [7,15]. Cohorts are not retrospectively relabeled using a different framework, and thresholds from the two systems are not combined. When findings are compared across definitions, the individual components of sarcopenia, including grip strength, muscle quantity or quality, and physical performance, should be reported wherever possible so that differences in case classification remain visible. SDOC is used as an outcomes-oriented comparator rather than as a substitute operational framework [6,15,25]. For clinical implementation, services should select the framework appropriate to their population and locally validated reference standards and apply it consistently from admission through follow-up [7,12].

Sarcopenia and frailty overlap but are not interchangeable. Sarcopenia is a muscle disease defined through strength, muscle quantity or quality, and physical performance, whereas frailty is a multisystem state of heightened vulnerability to stressors [7,15,37,38]. Weakness and slow gait contribute to both constructs, so a person may meet both definitions; however, frailty can occur without confirmed sarcopenia, and sarcopenia can occur without global frailty. A brief frailty measure, such as a validated phenotype, deficit-accumulation index, or Clinical Frailty Scale, can complement sarcopenia assessment by capturing cognition, comorbidity, exhaustion, and broader dependence, but it cannot replace muscle-specific testing when a muscle-focused clinical or research decision is required. In a time-limited service, frailty screening may serve as a first-pass risk assessment, followed by framework-specific sarcopenia testing when low strength, slow gait, weight loss, or poor recovery raises concern [22,37,38].

## 4. Mechanisms Linking Sarcopenia to Impaired Gait Recovery

The biological core of sarcopenia-related walking failure is low reserve under stress. Aging muscle shows anabolic resistance, meaning a reduced protein synthetic response to both dietary amino acids and resistance exercise [39,40]. Cuthbertson and colleagues demonstrated reduced anabolic sensitivity to essential amino acids in older muscle, while Breen and Phillips summarized how aging blunts the muscle protein synthetic response to feeding and exercise. Available mechanistic work suggests that aging can blunt load-induced anabolic signaling and reduce muscle protein synthesis after contraction, which may contribute to poorer recovery after acute illness or inactivity in older adults [39,40]. When this baseline biology is superimposed on illness, surgery, pain, bed rest, or reduced ambulation, disuse atrophy can occur rapidly, especially in older adults. Contemporary work on disuse in aging consistently identifies anabolic resistance, inflammation, mitochondrial stress, and proteostatic disruption as key amplifiers of muscle loss [41,42,43].

Inflammation is the other major amplifier. The acute sarcopenia model proposed by Welch and colleagues explicitly links hospitalization-related muscle decline to the combination of inflammatory burden and muscle disuse. In older adults, inflammatory markers have long been associated with poorer physical performance. In the other studies, higher IL-6 and CRP were related to poorer walking speed and grip strength, and more recent reviews continue to point to hs-CRP and IL-6 as candidate correlates of gait impairment and frailty-related mobility decline. This inflammatory biology is especially relevant after infection, trauma, or major surgery, when rehabilitation often begins before the catabolic phase has fully resolved [20,44]. From a gait biomechanics perspective, anabolic resistance and illness-related disuse reduce the ability to generate rapid lower-limb force during weight acceptance and push-off, which can appear clinically as slower gait speed, shorter step length, and reduced walking endurance [39,43,45]. When muscle quality deteriorates through myosteatosis and impaired contractile efficiency, the patient may preserve some limb mass but still lose propulsive power and the capacity to accelerate, climb, or recover after a perturbation [7,19]. Neuromuscular remodeling further reduces motor unit precision and rate of force development, which is clinically consistent with cautious turning, reduced adaptability, and greater instability when walking demands become more complex [45,46]. Trunk weakness may amplify these deficits by impairing control of the center of mass during turning and transitional tasks, thereby increasing reliance on compensatory strategies during rehabilitation [5,47].

Yet sarcopenia does not impair gait through muscle fiber biology alone. Neuromuscular aging alters motor unit number and discharge behavior, reducing the precision and rapidity with which force can be generated during stepping and balance recovery [45]. At the biomechanical level, trunk strength and endurance are also important. In mobility-limited older adults, trunk muscle attributes are associated with both balance and mobility performance, and systematic review evidence suggests that trunk muscle strength relates to static and dynamic balance, functional performance, and falls. This is clinically intuitive. Effective gait recovery requires the capacity not only to push forward, but also to stabilize the torso, transfer load, and control the center of mass during turns, transfers, and perturbations [46,47].

Psychological and cognitive modifiers add a second layer of gait vulnerability. Fear of falling and fear-related activity restriction are highly prevalent in older adults, especially in those with sarcopenia, prefrailty, and frailty, and they can create a self-reinforcing cycle of reduced activity, worsening weakness, and greater dependence. At the same time, the gait cognition literature shows that slowing gait often coexists with executive and processing speed deficits, and specific work in sarcopenia suggests that its association with cognitive impairment is driven mainly by slow gait speed. In post-fracture rehabilitation, newer data also suggest that sarcopenia and cognitive impairment may jointly impair one-year walking recovery. For sarcopenia-aware rehabilitation, this means that a slow walker is not merely weak. The patient may also be cautious, underconfident, cognitively overloaded, or poorly able to integrate sensory input while moving [48,49].

**Figure 1:**
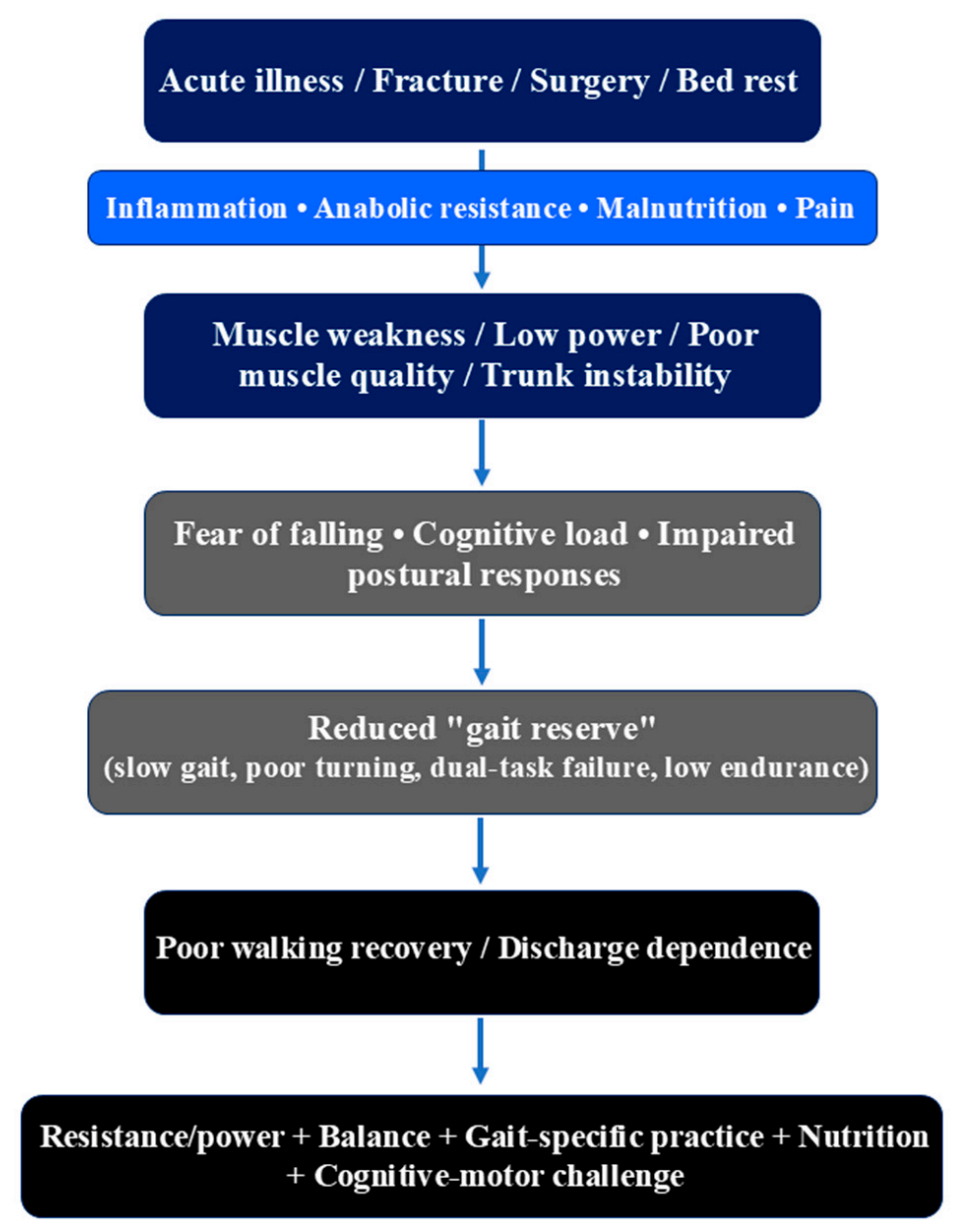
Proposed pathway linking acute illness, fracture, surgery, and deconditioning to impaired walking recovery through sarcopenia-related loss of gait reserve.

These observations explain why biomarkers and muscle quality markers are attracting growing attention. Muscle ultrasound is increasingly described as a patient-friendly, fast, low-burden bedside method that can capture both muscle size and qualitative change, and recent reviews support its correlation with reference measures and clinically relevant outcomes [36]. Echo intensity has been proposed as a marker of functional performance in older adults, while digital gait markers derived from dual-task walking, such as combined gait speed and turning metrics, may help identify sarcopenic and frailty-related gait phenotypes. None of these tools is yet ready to replace clinical assessment, but together they strengthen the case that sarcopenia-aware rehabilitation should measure reserve more intelligently than body mass estimation alone [19]. Figure 1 illustrates the proposed pathway linking acute illness, fracture, surgery and deconditioning to impaired walking recovery through sarcopenia-related loss of gait reserve.

## 5. Clinical Evidence Across Settings

In community-dwelling older adults with sarcopenia, exercise trials provide indirect evidence relevant to the design of gait-focused rehabilitation research, but they do not establish the effectiveness of a sarcopenia-aware approach in post-acute rehabilitation [24,27,28]. A 2023 systematic review and meta-analysis focused on locomotion concluded that exercise benefits walking-related outcomes in community-dwelling older adults with sarcopenia. Meta-analyses of resistance training have also found gains in grip strength, gait speed, and, in some analyses, skeletal muscle index, with more robust effects for strength than for morphology. Newer home-based studies likewise suggest that graded progressive resistance combined with aerobic activity can improve physical function, balance, and cardiopulmonary capacity, while exercise plus nutrition interventions in functional sarcopenia can improve gait speed, SPPB, grip strength, and quality of life even when changes in skeletal muscle index are modest. Together, these findings indicate that gait-related outcomes can improve in selected, relatively stable community samples; they should therefore be used to generate intervention hypotheses rather than to infer efficacy in medically complex rehabilitation inpatients (Table 1) [24,27,28]. 

Evidence from community-dwelling cohorts provides useful biological and rehabilitative signals, but it cannot be transferred uncritically to post-acute geriatric rehabilitation wards [27,28]. Compared with community participants, rehabilitation inpatients usually have greater multimorbidity, more pain, higher inflammatory burden, lower habitual activity, more cognitive fluctuation, and a narrower safety margin during mobility training, all of which may alter both sarcopenia measurement and response to treatment [5,10,20]. Accordingly, findings from community trials are interpreted in this review as indirect evidence that supports biologic plausibility and intervention design, not as direct proof of effectiveness in medically complex post-acute populations [8,10,24].

SPRINTT randomized 1519 community-dwelling adults aged 70 years or older with physical frailty and sarcopenia, SPPB scores of 3–9, low appendicular lean mass, and the ability to complete a 400 m walk [24]. In the prespecified SPPB 3–7 subgroup, mobility disability occurred in 46.8% of participants assigned to the multicomponent intervention and 52.7% assigned to healthy-aging education (hazard ratio 0.78, 95% confidence interval 0.67–0.92; absolute difference 5.9 percentage points). No benefit was demonstrated in the SPPB 8–9 subgroup (hazard ratio 1.25, 95% confidence interval 0.79–1.95). The result is clinically relevant but modest, subgroup-specific, and derived from community-dwelling participants rather than post-acute inpatients; it therefore supports feasibility and hypothesis generation, not direct transfer of efficacy to rehabilitation wards.

Orthogeriatric and post-hip-fracture rehabilitation adds both stronger urgency and greater complexity. A systematic review of older adults with hip fracture reported an overall sarcopenia proportion of about 44%, and most included studies found poorer postoperative functional recovery in patients with sarcopenia. Another study reported that sarcopenia negatively affected functional recovery among older patients admitted for in-hospital rehabilitation after hip fracture. Handgrip strength has also emerged as an important predictor in this setting: Savino and colleagues found that early grip strength evaluation provided prognostic information regarding future walking recovery, and Pérez-Rodríguez and colleagues later showed that lower grip strength predicted greater deterioration in walking ability and higher mortality one year after fracture [8,56].

The hip fracture evidence is not uniform. In the multicenter study by Steihaug and colleagues, sarcopenia was not associated with a change in the New Mobility Score from the prefracture estimate to one year (adjusted effect 0.2 points, 95% confidence interval −0.5 to 0.9; *p* = 0.6) [9]. Several design features may explain divergence from other cohorts: sarcopenia was assessed a median of four days after surgery using an EWGSOP1-era combination that included anthropometrically estimated muscle mass and retrospectively reported prefracture mobility; the change score was vulnerable to ceiling effects; rehabilitation exposure was not recorded; and participants unable to provide valid measurements or consent were underrepresented. Importantly, the sarcopenia group still had lower mobility at one year (mean New Mobility Score 5.8 versus 6.8; *p* = 0.003) and more death or nursing home residence. Thus, the study weakens a deterministic claim that sarcopenia predicts the magnitude of mobility change, but it does not negate its possible value as a marker of lower attained mobility and adverse disposition.

The practical implication is that sarcopenia status should neither be used to deny rehabilitation nor be presented as an individual prognosis. It may help identify patients who enter care with lower strength and mobility, but recovery targets and treatment dose should be based on serial performance, medical stability, cognition, pain, goals, and response to therapy. Future hip fracture studies should standardize assessment timing, use contemporary diagnostic frameworks, report rehabilitation exposure, and distinguish final mobility from change relative to prefracture function [8,9,51,56].

The medically complex and post-acute rehabilitation literature supports the same general direction, although it is less developed and more heterogeneous. In older in-hospital rehabilitation patients, a study found that a higher probability of sarcopenia was associated with worse overall functional status and poorer walking ability at discharge. Other rehabilitation cohort work has linked sarcopenia, low physical activity, and poor nutritional status with worse outcomes and higher post-discharge mortality, reinforcing the view that walking recovery in these settings is biologically entangled with muscle vulnerability [8,53].

Cardiac rehabilitation illustrates the distinction between prognosis and treatment. In adults aged 75 years or older entering inpatient rehabilitation after cardiac procedures, sarcopenia was present in 35% at baseline and was associated with poorer functional capacity [10]. Gait speed also has prognostic capability comparable with 6 min walk distance in older adults with cardiovascular disease [54]. These findings provide a potentially useful risk stratification signal, although residual confounding by cardiac disease severity, procedural burden, inflammation, and deconditioning remains likely. They do not demonstrate that a sarcopenia-specific intervention improves gait beyond contemporary cardiac rehabilitation [10,54,55].

A prognostic association should not be equated with treatment efficacy. Evidence that sarcopenia or low grip strength identifies a group with poorer mobility may justify closer monitoring and stratified research, but it does not establish that modifying a sarcopenia label or component changes walking recovery. Table 2 therefore reports confidence in prognostic or risk stratification use separately from confidence in treatment efficacy, using the appraisal domains defined in Section 2.

## 6. Muscle Strength and Muscle Quality as Clinically Informative Markers of Walking Recovery Risk

Current evidence suggests that muscle strength and muscle quality may be more clinically informative than muscle mass alone when discussing walking-related prognosis in older adults, although this conclusion remains provisional because diagnostic definitions, measurement tools, and study populations are heterogeneous [6,7,25].

In rehabilitation-relevant cohorts, low handgrip strength, slowness, and poor physical performance repeatedly track with adverse mobility outcomes, whereas lean mass alone shows a weaker and less consistent relationship with clinically meaningful recovery end points [14,25,28]. This pattern supports closer attention to strength and muscle quality in assessment, but it should not be interpreted as proof that any single strength-focused intervention will improve walking recovery across all post-acute populations [8,24].

Table 2 applies the prespecified narrative appraisal criteria described in Section 2 to each clinical setting and type of inference. Risk-of-bias judgments consider study design, participant selection, timing and validity of sarcopenia measurement, attrition, and residual confounding. Consistency reflects whether the direction of findings is similar across studies and outcomes. Directness considers whether the population, sarcopenia construct, rehabilitation setting, and walking outcome correspond to medically complex post-acute rehabilitation. Precision considers sample size, number of outcome events, and confidence intervals when these data are reported. The resulting confidence categories are qualitative narrative judgments rather than numerical scores or formal GRADE certainty ratings [30,57].

Bedside case findings should be proportionate to setting and purpose (Table 3). A pragmatic core bundle can combine SARC-F or SARC-CalF, standardized grip strength, usual-pace gait speed when safe, and a validated malnutrition screen. Services may target approximately 5–10 min by embedding these elements in routine nursing and therapy assessments, but actual time should be audited locally. A positive symptom screen should prompt objective testing rather than act as a diagnosis, and inability to walk safely should be documented rather than scored as sarcopenia by default [7,15,22,58].

The minimum training requirement is protocol-specific competency: standardized dynamometer position and commands for grip strength; a measured walkway, safety procedure, and documentation of assistive devices for gait speed; and instrument-specific instruction for nutrition screening. Chair stands and SPPB require clinical judgment about weight-bearing restrictions, pain, balance, and cognition. Muscle ultrasound, bioimpedance-derived phase angle, and DXA belong to an enhanced tier because they require equipment, calibration, trained operators, and reproducibility checks. They should not delay usual rehabilitation when unavailable [12,14,36,59]. Repeating the same feasible measures at discharge is more interpretable than changing tools during the episode of care.

**Table 3:** Candidate bedside tools for sarcopenia screening and gait-related risk stratification in older rehabilitation patients.

Tier	Tool	Approximate Time	Potential Owner	Interpretation and Role	Training, Resources, and Safety
Core	SARC-F or SARC-CalF	2–3 min	Nurse, PT, OT, or physician	Symptom-based case finding; a positive result should prompt objective assessment of strength and physical performance rather than be treated as a diagnosis [58,60].	Minimal training. SARC-F has limited sensitivity; calf circumference may be affected by edema, obesity, hydration, and measurement technique.
Core	Grip strength	3–5 min	Trained nurse, PT, OT, or physician	Identifies low muscle strength within the selected EWGSOP2 or AWGS 2019 framework [7,15].	Requires a validated dynamometer, standardized posture and commands, and documentation of laterality. Hand pain, arthritis, neurological impairment, and inability to follow commands may affect validity.
Core	Usual-pace 4 m gait speed	Usually <5 min	PT, OT, or appropriately trained nurse when safe	Provides a global mobility measure and longitudinal outcome. Apply the cutoff from the selected diagnostic framework and report the absolute speed [7,15,59].	Requires a measured walkway, standardized acceleration protocol, guarding when necessary, and documentation of footwear, assistance, and walking device. It is not specific to sarcopenia.
Conditional core	Five-chair-stand test or SPPB	5–10 min	PT, OT, or another trained clinician	Assesses lower-limb physical performance and contributes to severity classification when testing is safe and valid [7,15].	Clinical judgment is required when pain, weight-bearing restrictions, balance impairment, delirium, fatigue, or inability to follow commands is present.
Core	MNA-SF, MUST, or another validated malnutrition screen	3–5 min	Nurse or dietitian	Identifies nutrition risk and triggers comprehensive dietetic assessment; it does not diagnose sarcopenia [21,22,23].	Requires reliable weight and intake information. Positive findings should prompt assessment of swallowing, dentition, gastrointestinal symptoms, fluid status, and barriers to food access.
Enhanced	DXA	Local workflow-dependent; excludes transport	Trained imaging staff	Measures appendicular lean mass and may confirm low muscle quantity when required for framework-specific diagnosis, research eligibility, or follow-up [7,15].	Requires imaging equipment, calibration, transport, positioning, and population-appropriate cutoffs. It should not delay usual rehabilitation.
Enhanced	BIA, including optional phase angle output	Usually <10 min; protocol dependent	Trained clinical or research staff	Estimates muscle quantity when validated equations and framework-specific cutoffs are available. Phase angle is an exploratory adjunct and should not replace diagnostic muscle-quantity measures [7,12,15].	Results are affected by hydration, edema, recent intake, device type, electrodes, equations, and positioning. The same device and protocol should be used for serial assessment.
Enhanced	Muscle ultrasound	Commonly ≥10 min	Trained operator	Provides an exploratory assessment of muscle thickness, architecture, and echo intensity for research or advanced clinical services [19,36].	Requires equipment, standardized anatomical landmarks, device settings, positioning, operator training, and documented intra- and inter-rater reliability. It is not currently a routine substitute for EWGSOP2 or AWGS muscle-quantity criteria.

Footnotes: Reported completion times are approximate and exclude transport, equipment preparation, documentation, and delays related to medical instability. Potential owners should be adapted to local scope-of-practice requirements and demonstrated competency. A positive case finding result is not equivalent to confirmed sarcopenia, and inability to complete a walking or chair-stand test safely should be documented rather than automatically classified as poor performance. Services should select either EWGSOP2 or AWGS 2019 a priori and should not combine thresholds from different frameworks. Enhanced tools should not delay usual rehabilitation when unavailable. Abbreviations: AWGS, Asian Working Group for Sarcopenia; BIA, bioelectrical impedance analysis; DXA, dual-energy X-ray absorptiometry; EWGSOP2, European Working Group on Sarcopenia in Older People 2; MNA-SF, Mini Nutritional Assessment–Short Form; MUST, Malnutrition Universal Screening Tool; OT, occupational therapist; PT, physical therapist; SPPB, Short Physical Performance Battery.

## 7. Multicomponent Rehabilitation: A Promising but Incompletely Tested Strategy

Multicomponent rehabilitation is biologically plausible and increasingly supported by indirect evidence, particularly when programs combine strengthening, balance challenge, and task-specific mobility practice [24,27,28]. However, the available evidence is still more robust in community-based samples than in medically complex inpatient rehabilitation populations, so this approach should be framed as a promising clinical direction rather than an established standard of care across settings [5,9,24].

The strongest conceptual support for this hypothesis comes from interventions that combine physical domains instead of isolating them. In SPRINTT, the multicomponent intervention produced a modest reduction in mobility disability only in the prespecified SPPB 3–7 subgroup of community-dwelling participants; the finding therefore provides indirect, hypothesis-generating support rather than evidence of efficacy in post-acute rehabilitation [24]. In older adults at high fall risk, treadmill training augmented with virtual reality reduced fall rates compared with treadmill training alone, indicating that gait can be improved more effectively when cognitive motor challenge is added to motor practice [24,61]. Reviews of dual-task exercise likewise suggest benefits for balance and gait, and frailty-focused cardiac rehabilitation studies report that adding resistance and balance training can more effectively normalize slow gait speed than standard cardiac rehabilitation alone. Trunk-focused programs also show consistent associations with better balance and functional performance, which is relevant because many sarcopenic walkers fail during turning, obstacle negotiation, or transitional movements rather than during straight-path walking only [24,47].

Taken together, the indirect evidence suggests that rehabilitation combining progressive resistance or power training, balance retraining, trunk stabilization, task-specific gait practice, and cognitive motor challenge may improve gait-related outcomes, but this remains to be tested directly in medically complex post-acute populations. This hypothesis is especially plausible for patients whose primary failure mode is low gait reserve. Such patients may look adequate on a short straight walk, yet fail when walking becomes fast, prolonged, cognitively loaded, or environmentally variable. Whether an integrated program targeting these reserve-dependent tasks provides additional benefit beyond individualized usual rehabilitation remains a hypothesis for prospective testing [24,27,28,61]. Future randomized trials should therefore compare usual therapy with integrated programs using outcomes that reflect both impairment and participation, including gait speed, TUG, SPPB, 6 min walk distance, Koval or FAC ambulation, falls, ADL and, where possible, life-space mobility [27,61,62].

## 8. Nutrition Assessment and Individualized Support During Recovery

Nutrition care is clinically important because malnutrition, low intake, inflammation, and prolonged bed rest can limit rehabilitation tolerance. Its strongest justification in post-acute care is the identification and treatment of malnutrition, inadequate intake, and reversible deficiencies, not an assumption that a supplement prescribed on the basis of sarcopenia alone will restore gait [20,21,22,23,63,64]. Nutrition should therefore be presented as supportive, individualized care delivered alongside rehabilitation, with gait-specific efficacy remaining uncertain.

A practical workflow begins with a validated malnutrition screen at admission, followed by comprehensive dietetic assessment when risk is identified. Assessment should include recent weight loss, body mass index, current intake, appetite, swallowing and dentition, gastrointestinal symptoms, renal and hepatic function, inflammation or disease burden, food access, and cultural preferences. GLIM criteria may support formal malnutrition diagnosis after screening, but they do not replace a full nutrition assessment [21,23]. The team should set individualized energy and protein goals, document meal and oral nutrition supplement intake, coordinate nutrition with therapy schedules, and reassess weight, intake, tolerance, and functional at clinically appropriate intervals during inpatient rehabilitation.

Guideline-based protein targets require clinical individualization. At least 1.0 g/kg/day is generally advised for older adults; approximately 1.0–1.2 g/kg/day is often proposed for healthy older people and 1.2–1.5 g/kg/day during acute or chronic illness, with higher intakes considered only in selected severe illness, injury, or malnutrition under clinical monitoring [21,22,23,64]. Energy adequacy is necessary for protein to be used effectively. A practical pattern is approximately 25–30 g of high-quality protein at each main meal, adjusted for body size, usual diet, overall tolerance, and conditions that require protein restriction or modification, particularly advanced kidney or liver disease. Evidence in post-acute sarcopenia does not establish that pre-exercise dosing is superior to post-exercise dosing. Total daily intake and adherence should take priority; offering a protein-rich meal or supplement near the therapy session, including within approximately two hours afterward, is a pragmatic option rather than a proven anabolic window [21,22,23,64].

Vitamin D deficiency should be identified and treated according to local laboratory thresholds and prescribing guidance, while routine high-dose supplementation without documented deficiency is not supported as a gait recovery strategy. Iron, vitamin B12, folate, and other micronutrients should likewise be corrected when deficiency or a credible clinical indication is present rather than prescribed as a universal sarcopenia regimen [22,23]. Common barriers include anorexia, early satiety, dysphagia, poor dentition, delirium, nausea, constipation, dietary restrictions, gastrointestinal intolerance, cost, and limited help with meals. Responses may include food fortification, preferred protein-rich foods, texture modification after swallowing assessment, smaller frequent meals, oral nutrition supplements, symptom treatment, mealtime assistance, and social work support. Dietitians, nurses, speech language therapists, physicians, pharmacists, and rehabilitation clinicians should share explicit responsibilities.

The 2023 meta-analysis by Song and colleagues included 12 trials and 713 older adults with sarcopenia who were generally ambulatory or stable enough to undertake resistance training [28]. Nutritional supplementation added to resistance training did not significantly improve gait speed overall (standardized mean difference 0.17; 95% confidence interval, −0.03 to 0.36; *p* = 0.098). A protein-plus-vitamin D subgroup showed a small effect (standardized mean difference 0.36, 95% confidence interval 0.08–0.63; *p* = 0.011), but formulations were heterogeneous, protein doses ranged from approximately 13.5 to 35 g per administration, and all groups received exercise. The analysis did not establish a dose–response relationship, isolate an exercise-independent nutrition effect, or permit conversion of the standardized effect into a clinically meaningful change in m/s. It should not be generalized directly to post-acute inpatients. Other combined exercise and nutrition studies reported improvements in gait speed, SPPB, grip strength, or instrumental activities of daily living despite modest changes in skeletal muscle index [65,66]. These studies do not isolate the independent effect of nutrition and were conducted mainly in community-dwelling participants who were sufficiently stable to undertake structured exercise.

The broader protein supplementation literature reinforces this caution. An overview of 33 systematic reviews covering 441 unique studies found no consistent improvement in physical performance among older adults overall or among healthy older adults. Small gains in muscle mass and strength were more apparent in older adults with long-term conditions, particularly when protein supplementation accompanied exercise; however, the evidence for improved physical performance in these groups was low-certainty, and no performance effect was established in hospitalized patients. Among hip fracture inpatients, supplementation reduced medical complications but did not establish improved gait recovery [67]. These findings support targeted correction of inadequate intake and malnutrition rather than universal supplementation based solely on a sarcopenia label.

**Figure 2:**
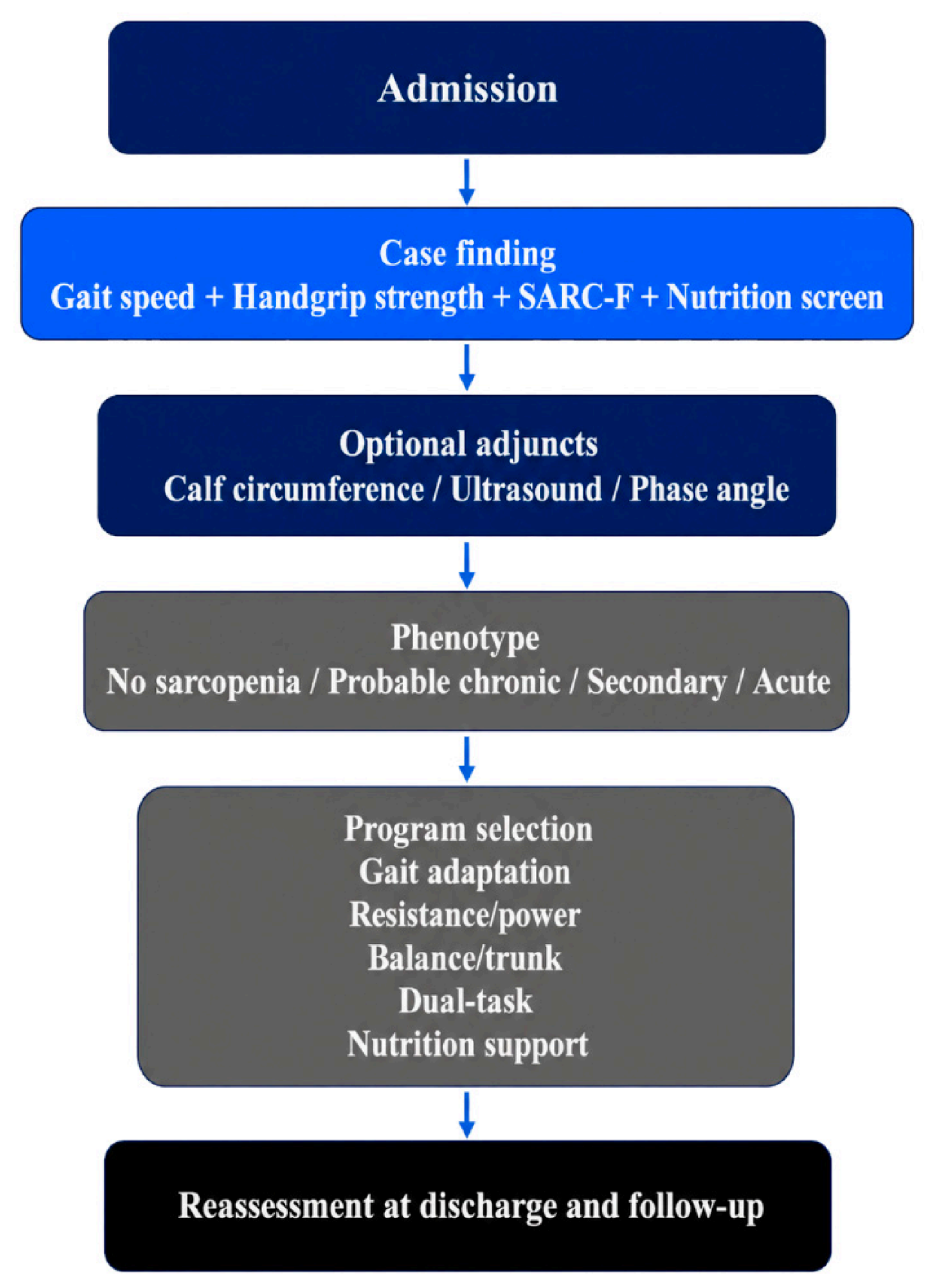
Hypothesis-generating admission-to-discharge framework for sarcopenia-aware assessment and risk-informed gait care. The core tier is designed for routine post-acute settings; enhanced measures are optional and should not delay usual rehabilitation.

Direct gait-specific nutrition trials in medically complex post-acute rehabilitation populations remain scarce, despite the high prevalence of bed rest, inflammation, multimorbidity, poor appetite, and medication burden in these settings [5,7,20,34]. When malnutrition risk or inadequate intake is identified, guideline-concordant nutrition assessment and treatment are appropriate components of comprehensive post-acute care [21,22,23,63,64]. Their purpose is to address malnutrition, reversible deficiencies, and barriers to rehabilitation participation; current evidence does not establish that nutrition care alone, or treatment directed specifically at sarcopenia, improves gait recovery in medically complex post-acute inpatients [62,65,66,68]. Figure 2 integrates these principles: core case findings include gait speed, grip strength, SARC-F, and a nutrition screen; positive findings lead to framework-specific diagnostic clarification and individualized rehabilitation, while nutrition care is escalated when malnutrition risk or inadequate intake is confirmed. Enhanced measures are optional and should not delay usual rehabilitation. Because the pathway is conceptual, its feasibility, incremental value, and effect on walking outcomes require prospective evaluation [7,15,22,59].

Implementation depends on more than selecting tests. Institutions need a brief written protocol that defines the target population, chosen diagnostic framework, test ownership, contraindications, documentation fields, referral thresholds, and reassessment schedule. A minimum viable pathway can be embedded in existing nursing, therapy, medical, and dietetic assessments, with one discipline accountable for closing the loop. Short interdisciplinary huddles and shared electronic templates can reduce duplicate testing. Personnel shortages may require cross-training and a core bundle that can be completed without specialist imaging; ultrasound, phase angle, and DXA should be added only when equipment, calibration, operator competency, and quality assurance can be sustained [52,59].

Funding and reimbursement vary by jurisdiction. The United States ICD-10-CM code M62.84 recognizes sarcopenia diagnostically, but a diagnostic code does not guarantee payment for a stand-alone screen, imaging test, supplement, or rehabilitation service [69]. Services should verify local payer rules and, where appropriate, document case findings within medically necessary comprehensive geriatric, rehabilitation, and nutrition assessments rather than assume universal reimbursement. Before wider adoption, institutions should audit completion time, missingness, inter-rater reliability, referrals generated, treatment changes, patient burden, equity, and walking outcomes. These implementation measures are prerequisites for evaluating whether the proposed framework adds value beyond routine care [59,69].

## 9. Strengths and Limitations of the Current Evidence

The evidence sources in this review do not support equal clinical inference. Repeated associations between weakness, slowness, poor physical performance, and adverse mobility outcomes support further prognostic investigation, whereas direct evidence that treatment directed by sarcopenia status improves post-acute walking remains sparse [6,7,14,25]. Across the included evidence, risk of bias arises primarily from residual confounding, selective measurement after acute illness, attrition, retrospective estimation of pre-event function, and heterogeneous diagnostic and outcome definitions. Consistency is moderate for associations with poorer baseline or attained mobility but weaker for change in mobility over time, as illustrated by the discrepant hip fracture findings [8,9,51,56]. Directness is limited when community-based intervention trials are extrapolated to medically complex rehabilitation inpatients, and precision is frequently constrained by small cohorts, few gait-specific outcomes, or incompletely reported confidence intervals (Table 2).

This mini-review also has important methodological limitations. The literature search was targeted rather than systematic, study selection was not performed independently by two reviewers, and a formal study-level risk-of-bias assessment using design-specific instruments was not undertaken. Instead, the evidence was evaluated through a structured narrative appraisal at the setting-and-claim level, and the confidence categories should therefore not be interpreted as formal GRADE ratings. Relevant studies may have been missed, and the review cannot provide pooled effect estimates or definitive comparative effectiveness conclusions. Nevertheless, a strength of the review is its explicit separation of prognostic or risk-stratification evidence from treatment efficacy evidence, together with its setting-specific assessment of risk of bias, consistency, directness, and precision. The synthesis therefore identifies where prognostic signals are reasonably supported and where treatment claims remain untested.

## 10. Future Directions

The points below are framed as research priorities rather than current standards of care [5,24]. First, future studies need a more consistent diagnostic framework and should report the individual components of sarcopenia separately, including strength, muscle quantity or quality, and physical performance, because these components do not behave identically across settings [7,15,33]. Second, post-acute and medically complex rehabilitation populations remain insufficiently studied, so gait-specific trials in these settings should be considered hypothesis-testing work rather than extensions of an already settled evidence base [5,10].

Third, implementation science is now needed as much as mechanistic science. Sarcopenia is often underdocumented in rehabilitation populations, even when it is prevalent, and bedside methods that are feasible, predictive, and scalable still need validation in real clinical workflows. Research priorities should include pragmatic admission triage bundles, biomarker-enriched cohorts, ultrasound-enabled muscle quality monitoring, and comparative trials that test whether strength-informed program design changes therapist decision-making, dose progression, and outcomes in ordinary services rather than only under ideal experimental conditions [52].

## 11. Open Questions and Limitations

Unresolved questions include which sarcopenia framework performs best in different rehabilitation populations, whether challenged walking adds prognostic information beyond usual gait speed, which combination and dose of resistance, balance, gait, and nutrition care is most effective, and whether technology-enabled muscle quality measures provide sufficient incremental value to justify their cost. The inconsistent hip fracture findings further show that sarcopenia is not a deterministic barrier to recovery [46]. Future work should distinguish prediction of final mobility from prediction of change and should examine whether frailty assessment adds value, serves as a first-pass screen, or identifies a different treatment-relevant phenotype [22,37,38].

## 12. Conclusions

Current evidence shows that weakness, slowness, and poor muscle quality associated with sarcopenia are clinically relevant in older adults with mobility impairment, but these factors alone do not determine walking recovery. Current observational evidence suggests that sarcopenia-related weakness and slowness may help identify a vulnerable rehabilitation phenotype after hospitalization, hip fracture, or cardiac procedures, but their prognostic value beyond disease severity, frailty, and baseline mobility remains uncertain. However, no single sarcopenia-aware rehabilitation approach has yet been proven superior across post-acute settings, and it would be premature to promote any uniform package of screening and interventions as standard care. Going forward, well-designed trials are needed to determine whether early strength-focused assessment, nutrition-informed care, and multicomponent gait rehabilitation genuinely improve walking outcomes in medically complex older adults beyond what is achieved by current rehabilitation practices.

## Figures and Tables

**Table 1:** Summary of clinical evidence linking sarcopenia constructs with walking-related outcomes across rehabilitation-relevant settings.

Setting	Population	Sarcopenia Marker(s)	Mobility Outcomes	Evidence Direction	Main Limitations
Community-dwelling	Older adults with sarcopenia, frailty, or low physical performance	Low strength, slow gait, low lean mass, or low SPPB	Gait speed, TUG, SPPB, 6MWD, and mobility disability	Exercise programs generally improve gait speed and lower-limb performance [24,27,28].	Evidence is indirect for medically complex post-acute patients [24,27,28].
Hip fracture and orthogeriatrics	Older adults after hip fracture surgery	Sarcopenia diagnosis, low grip strength, or poor physical performance	Walking recovery, balance, ambulation, function, and mortality	Sarcopenia and weak grip are usually associated with poorer attained mobility [8,50,51].	Assessment timing, definitions, and rehabilitation exposure vary [8,50,51].
Geriatric post-acute rehabilitation	Medically complex rehabilitation inpatients	Probable sarcopenia, low grip strength, or poor physical performance	ADL, discharge walking, independence, and institutionalization	Low strength or probable sarcopenia is associated with poorer discharge function [14,52,53].	The observational evidence base is small, and acute illness may distort testing [14,52,53].
Cardiac rehabilitation	Older adults after cardiac procedures or acute heart failure	Sarcopenia, low grip strength, low SPPB, or slow gait	Functional capacity, gait speed, 6MWD, and rehabilitation response	Sarcopenia is associated with poorer baseline capacity; treatment response evidence is limited [9,54,55].	Cardiac severity and deconditioning may confound associations [9,54,55].

**Table 2:** Setting-specific structured narrative appraisal of evidence linking sarcopenia with walking recovery. Risk of bias, consistency, directness, precision, and confidence by inference type.

Setting	Risk of Bias/Quality	Consistency	Directness	Precision	Overall Confidence and Interpretation
Community-dwelling older adults	Randomized trials and systematic reviews are available, although selection, adherence, lack of blinding, and performance bias remain relevant.	Functional benefit is generally consistent, but effect sizes and intervention components vary [24,27,28].	Direct for relatively stable community-dwelling participants; substantially indirect for medically complex post-acute inpatients.	Several large trials and meta-analyses are available, but estimates vary and subgroup findings are not uniformly precise.	Moderate for exercise-related functional benefit in selected community populations; low for direct transfer to post-acute rehabilitation.
Hip fracture and orthogeriatrics	Evidence is predominantly observational and is affected by residual confounding, retrospective prefracture function, variable assessment timing, attrition, and heterogeneous sarcopenia definitions.	Most studies associate sarcopenia or low grip strength with poorer attained function, but findings for change in mobility are inconsistent, including the null Steihaug result [8,9,51,56].	The population is directly relevant, but rehabilitation exposure and gait-specific outcomes are inconsistently reported.	Many cohorts are modest in size, and confidence intervals or adjusted estimates are not consistently reported.	Low-to-moderate for prognosis or risk stratification; low for sarcopenia-specific treatment efficacy.
Geriatric post-acute rehabilitation	Observational cohorts are vulnerable to acute illness confounding, selective measurement, missing data, and misclassification when patients cannot complete performance tests.	Available studies generally indicate poorer discharge function with low strength or probable sarcopenia, but the evidence base is small [14,52,53].	Directly relevant population, although outcomes are often broad measures of function rather than gait-specific recovery.	Precision is limited by relatively small cohorts, incomplete reporting of confidence intervals, and heterogeneous outcomes.	Low-to-moderate for prognosis or risk stratification; low for treatment efficacy.
Cardiac rehabilitation	Prospective cohorts and secondary analyses remain susceptible to confounding by cardiac disease severity, procedural burden, inflammation, and deconditioning.	Available studies consistently identify lower baseline functional capacity, but gait-specific recovery findings are sparse [10,54,55].	Direct for older cardiac rehabilitation populations, but only partly direct for sarcopenia-specific walking recovery.	Few gait-specific estimates and no targeted sarcopenia-treatment trial are available.	Low-to-moderate for prognosis or entry risk stratification; low for treatment efficacy.

## Data Availability

No new data were created or analyzed in this study. Data sharing is not applicable to this article.

## Abbreviations

The following abbreviations are used in this manuscript:

6MWD	Six-minute walk distance
ADL	Activities of daily living
AWGS	Asian Working Group for Sarcopenia
EWGSOP2	European Working Group on Sarcopenia in Older People 2
FAC	Functional Ambulation Categories
hs-CRP	High-sensitivity C-reactive protein
IL-6	Interleukin-6
mTORC1	Mechanistic target of rapamycin complex 1
RESORT	REStORing health of acutely unwell adulTs
SARC-F	Strength, Assistance with walking, Rise from a chair, Climb stairs, and Falls questionnaire
SDOC	Sarcopenia Definitions and Outcomes Consortium
SPPB	Short Physical Performance Battery
SPRINTT	Sarcopenia and Physical fRailty IN older people: multi-componenT Treatment strategies
TUG	Timed Up and Go
